# Human Y chromosome pan-organ mapping reveals progressive mosaic loss from normal to cancer

**DOI:** 10.1172/jci.insight.201996

**Published:** 2026-09-08

**Authors:** Arkadiusz Gertych, Huihui Ye, Xingyu Chen, Eric Vail, V. Krishnan Ramanujan, Lauren Brady, Lawrence D. True, Peter S. Nelson, Peter R. Carroll, Dan Theodorescu

**Affiliations:** 1 Department of Surgery,; 2 Department of Pathology and Laboratory Medicine,; 3 Department of Urology, and; 4 Department of Biomedical Sciences, Cedars-Sinai Medical Center, Los Angeles, California, USA.; 5 Fred Hutchinson Cancer Center, Seattle, Washington, USA.; 6 Department of Laboratory Medicine and Pathology, University of Washington, Seattle, Washington, USA.; 7 Department of Urology, UCSF, San Francisco, California, USA.; 8 University of Arizona Comprehensive Cancer Center, Tucson, Arizona, USA.

**Keywords:** Clinical Research, Genetics, Oncology, Biomarkers, Molecular pathology, Urology

## Abstract

BACKGROUND. Loss of the Y chromosome (LOY) is a frequent event in male tumors and has been linked to cancer progression. However, the degree of mosaic LOY (mLOY) within normal tissues from men with or without cancer remains uncharacterized.

METHODS. Here we used a FISH-based assay targeting X- and Y-chromosome centromeres to perform a pan-organ analysis of mLOY in 1,000 male tissue samples from 405 individuals representing 11 organs. Automated image processing generated a quantitative FISH-based mLOY score (YchrFISH) that we validated against a transcriptomic surrogate of Y-chromosome dosage from RNA-seq data.

RESULTS. mLOY burden varied by tumor type, with highest degree in colorectal carcinoma. Across tissue groups, YchrFISH scores declined progressively from normal tissues of cancer-free men to histologically normal tissues adjacent to cancer and carcinoma (*P* < 0.0001). Paired analyses confirmed consistently greater mLOY in malignant compared with tumor-adjacent histologically normal tissue in different organs. Spatially resolved RNA-seq maps of bladders removed for cancer demonstrated a transcriptional gradient of Y-chromosome loss from normal urothelium through intraepithelial neoplasia to invasive carcinoma.

CONCLUSION. mLOY gradients exist across histologically normal and malignant tissues, consistent with the concept of field cancerization. Our findings support epithelial mLOY as a biomarker of early malignant transformation and, to our knowledge, a previously unrecognized hallmark of male oncogenesis.

FUNDING. NIH grants R35CA294022, P01CA163227, and P50CA97186 (the Pacific Northwest Prostate Cancer SPORE) and the Institute for Prostate Cancer Research.

## Abstract 2

Y chromosome loss increases progressively from healthy tissues to cancer, suggesting it is an early and previously unrecognized driver of cancer development.

## Graphical abstract

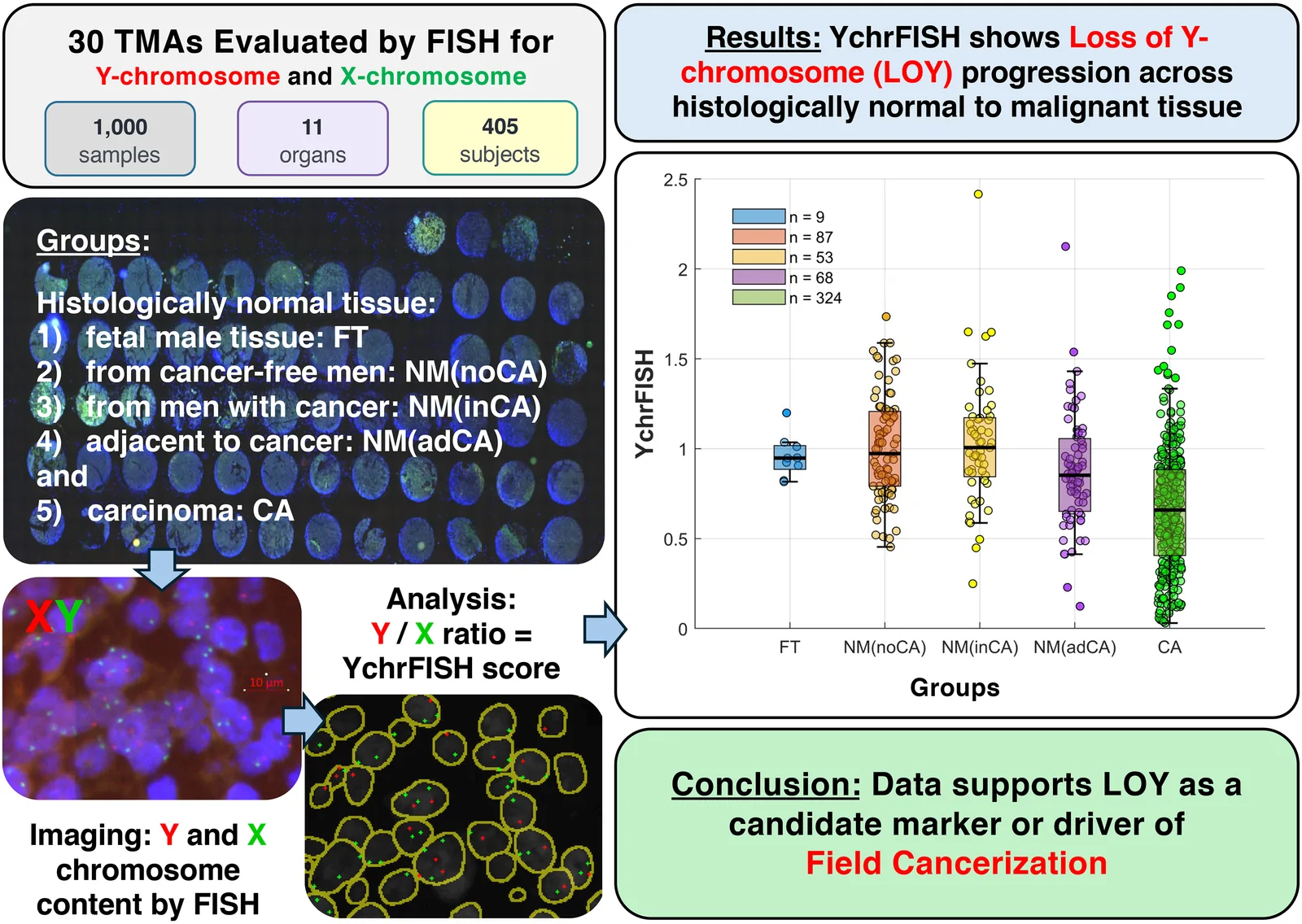

## Introduction

Loss of the Y chromosome (LOY) in a certain proportion of peripheral blood mononuclear cells (PBMCs) (1), termed mosaic LOY (mLOY) (2), is the most common genomic alteration in aging males and is linked to increased incidence and mortality in several cancer types, including lung, prostate, colon, and bladder (3–7). mLOY has also been observed in cancer cells and its degree varies in different tumor types, with the highest seen in papillary renal cell carcinoma (80%) and esophageal cancer (57%) and the lowest (<2%) seen in pheochromocytoma, paraganglioma, and thymoma (8, 9). While mLOY was extensively studied in blood cells and tumor tissue in pan-cancer genomic analyses, the degree of mLOY in normal tissue and premalignant lesions has not been systematically studied across different types of tissue.

Several studies have been performed to evaluate the degree of mLOY in benign tissues in various organs. A study of 277 samples of histologically normal urothelium and 145 bladder urothelial carcinoma showed that mLOY identified using single-nucleotide polymorphism microarray is found in 18% of normal urothelium and 29% of tumors (10). mLOY was also observed using fluorescence in situ hybridization (FISH) in benign hyperplastic prostatic epithelium in 8 out of 17 samples of prostate cancer exhibiting mLOY in the malignant epithelium of the same patient (11). A study using FISH detected concordant mLOY in ductal carcinoma in situ (CIS) adjacent to invasive breast cancer in 17 out of 22 male patients (12). However, little is known about the degree of mLOY in normal tissues from patients without a cancer history and normal tissue uninvolved with cancer, from cancer patients. Here we test the hypothesis that mLOY is an early event in cancer development, detectable across multiple organs in histologically normal tissues of cancer patients. We do this through an analysis of mLOY across 1,000 samples of normal tissues from men with or without cancer and malignant epithelium of different tissue types.

## Results

### Construction of a multiorgan tissue microarray repository.

We assembled a cohort of 2,917 formalin-fixed, paraffin-embedded (FFPE) tissue cores from males spanning fetal development to late adulthood (age range: 0.4–101 years). These cores were drawn from 7 institutional and commercial sources and categorized into 5 groups: fetal tissue (gestational age range: 0.4–0.5 years) (FT), histologically normal tissue from cancer-free men [NM(noCA)], histologically normal tissue from men with cancer in another tissue [NM(inCA)], histologically normal epithelium immediately adjacent to cancer [NM(adCA)], and carcinoma (CA) itself. The slide-level breakdown is the following: Cedars-Sinai autopsy multiorgan (CSMC-A, 4 slides; 645 cores), commercial multiorgan (COM-M, 6 slides; 431 cores), commercial fetal multiorgan (COM-FT, 1 slide; 57 cores), University of Virginia multiorgan (UVA, 2 slides; 284 cores), University of California San Francisco prostate (UCSF, 6 slides; 613 cores), Cooperative Human Tissue Network prostate/colon (CHTN, 2 slides; 220 cores), and Cedars-Sinai Medical Center bladder (CSMC-B, 9 slides; 667 cores). The samples covered 11 major organs, including bladder, prostate, kidney, liver, pancreas, stomach, colon, rectum, lung, esophagus, and head-and-neck regions from 405 individuals (Table 1).

### Validation of the mLOY assay.

**Figure 1:**
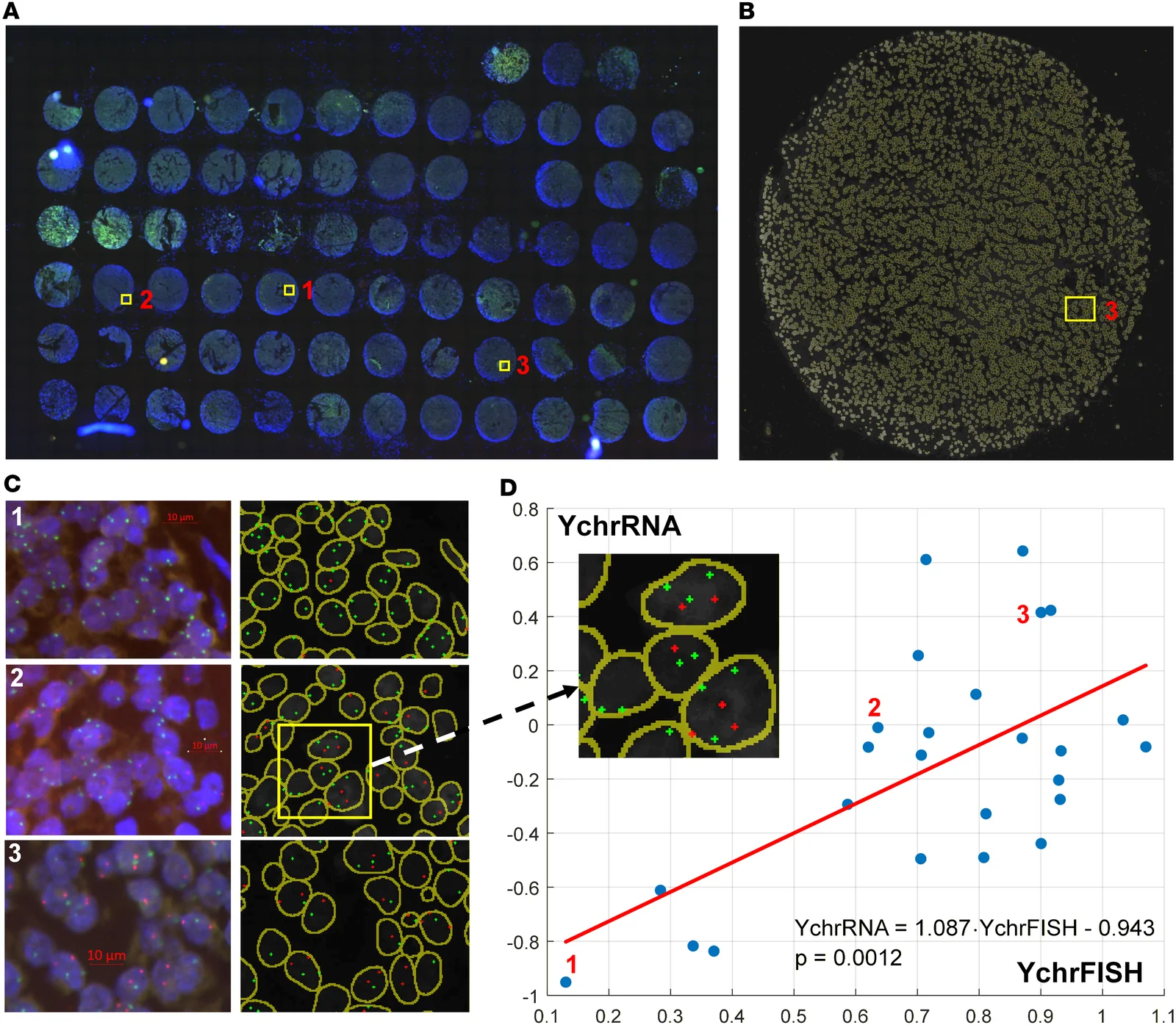
Positive correlation between FISH-based chromosome Y score (YchrFISH) and bulk RNA-seq–based chromosome Y score (YchrRNA) in paired TMA and frozen tumor samples of metastatic prostate cancer. (**A**) FISH-stained TMA (1–3 cores per site). Boxes represent areas used as example in **C**. (**B**) Low magnification of 1 representative core with automatically detected and delineated nuclei and detected FISH spots. (**C**) Micrographs of example FISH staining of 3 representative cores of soft tissue: liver (1), lymph node (2), and omental (3) metastases (left) with corresponding FISH spots detected and nuclei delineated (right). Scale bars: 10 μm. (**D**) Linear regression modeling (red line) indicates a statistically significant fit (*P* = 0.0012) between the YchrRNA and YchrFISH scores (*n* = 25, Pearson’s *r* = 0.61). Significance was assessed using a *t* test. Chromosomes Y and X detected in nuclei are marked orange and green, respectively.

The AneuVysion Multicolor DNA Probe Kit, a dual-color FISH assay targeting the centromeres of the X and Y chromosomes (13, 14), was used to detect X and Y chromosomes on tissue microarray (TMA) sections. Stringent image quality control based on contrast-to-noise ratio (CNR) metrics (15, 16) was then applied to DAPI, X-probe (green), and Y-probe (orange) fluorescence channels on 2,917 cores (see *Core image quality control* in Methods). Of these, 1,000 cores (34.3% of 2,917) comprising 226 cores from CSMC-A (35% of that source), 56 from COM-M (13%), 9 from COM-FT (16%), 129 from UVA (45%), 105 from UCSF (17%), 41 from CHTN (19%), and 434 from CSMC-B (65%) passed quality control and were retained for mLOY analysis. Next, we implemented an automated pipeline for image processing of these 1,000 cores; nuclei were segmented using the StarDist neural network model, and then FISH signals were identified using the fast radial symmetry (FRST) detector (17, 18). The number of nuclei per core ranged from 102 to 23,598, totaling 4,327,315 nuclei from 1,000 cores. These signals were used to generate a quantitative FISH-based mLOY score (YchrFISH) for each core defined as the ratio of orange (Y-probe) to green (X-probe) spots detected by FRST across all nuclei in each core image (Figure 1, A–C), in essence, the Y/X spot ratio across all nuclei.

To validate the YchrFISH quantification method, we compared YchrFISH scores of 25 metastatic prostate cancer specimens with a transcriptomic surrogate, the Y-chromosome expression score (YchrRNA) calculated using previously described methodology applied on RNA-seq, computed from bulk RNA-seq of matched OCT-frozen tumor samples (19). We observed a strong correlation (Pearson’s *r* = 0.61, *P* = 0.0012), indicating that in the paired tissue samples, the YchrFISH score closely tracks with Y-chromosome dosage at the transcriptional level reflected by YchrRNA score (Figure 1D).

### LOY is highly variable across tumor types.

**Figure 2:**
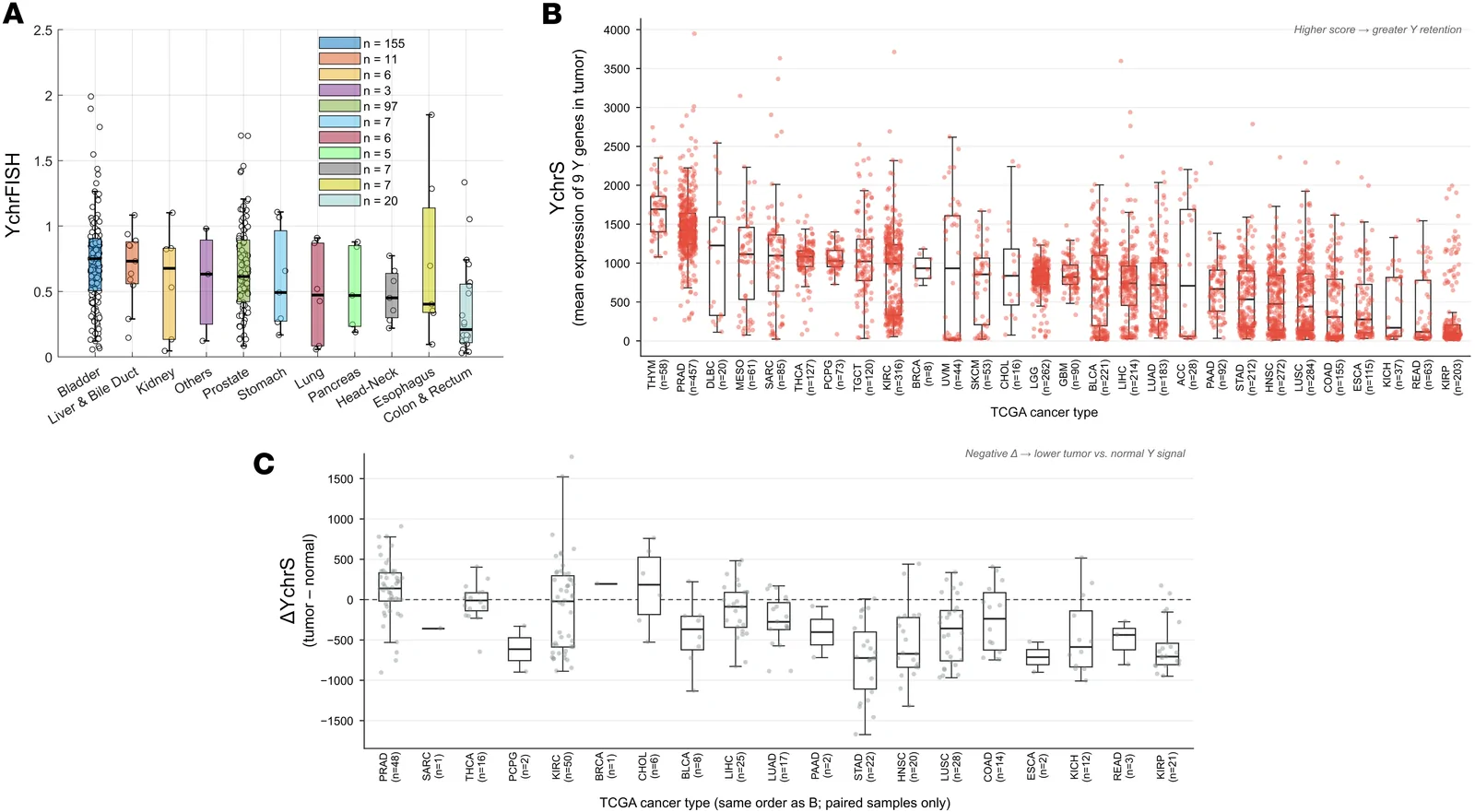
Degree of Y chromosome loss across male-derived cancer and normal tissues from different organs. (**A**) YchrFISH scores across multiple cancer types. One data point represents 1 individual. Cancer types are ordered by descending median YchrFISH score. (**B**) Absolute Y-chromosome RNA signature (YchrS) in male-proxy primary TCGA tumors. Each dot represents 1 tumor sample; boxes show the interquartile range and median. Cancer types are ordered from high to low median tumor YchrS to facilitate comparison with YchrFISH in **A**. Higher scores indicate greater Y-chromosome RNA signal, corresponding to less inferred Y loss. Sample sizes are shown under each cancer-type label. Across 28 cancer types, the highest median tumor YchrS was observed in thymoma (THYM), prostate adenocarcinoma (PRAD), and diffuse large B cell lymphoma (DLBC), and the lowest in kidney chromophobe (KICH), rectum adenocarcinoma (READ), and kidney renal papillary cell carcinoma (KIRP), among 3,869 primary tumors total. (**C**) Paired ΔYchrS, calculated as tumor minus matched normal tissue, for cancer types with tumor-normal pairs from the same patient. Gray dots represent individual paired patients; the dashed horizontal line marks zero. Negative ΔYchrS values indicate lower Y-chromosome RNA signal in tumor relative to matched normal. Only cancer types with paired normal tissue are shown; *n* denotes the number of paired patients. Cancer-type order matches **B** where applicable. Statistical testing for **C** is provided in the Supporting Data Values file using paired Wilcoxon signed-rank tests per cancer type.

The degree of mLOY, as measured by the YchrFISH score, varied markedly across 324 carcinoma samples from 11 distinct tissue types (Figure 2A). A higher YchrFISH score indicates a higher retention rate of Y chromosome and thus less mLOY. The lowest levels of mLOY (highest median YchrFISH scores) were observed in bladder (median score ≈ 0.75), hepatobiliary, (≈ 0.73), and kidney (≈ 0.67) cancers. In contrast, stomach (≈ 0.49), lung (≈ 0.47), pancreas (≈ 0.23), head-and-neck (≈ 0.45), esophageal (≈ 0.34), and colorectal (≈ 0.20) cancers displayed the lowest Y-chromosome signals, indicating the most substantial mLOY. Intermediate scores were seen in prostate cancer (≈ 0.61). These data indicate that mLOY is not uniform across cancer but instead reflects tissue-specific patterns.

To compare this tissue-specific ranking, we analyzed RNA-seq–based mLOY scores (YchrS) derived from the paired tumor and normal samples in The Cancer Genome Atlas (TCGA). Figure 2B ranks 28 cancer types according to the median tumor YchrS score across 3,869 tumor samples from 3,868 unique patients (one patient had 2 primary tumor samples). For the 298 tumor-normal sample pairs with known cancer type, we then calculated ΔYchrS as the absolute difference between tumor and matched normal YchrS scores. Additional 87 pairs lacking cancer type information were not included in this analysis. The median ΔYchrS was negative in 16 of 19 cancer types with paired samples (Figure 2C), indicating lower YchrS in tumors than in matched normal tissue. This decrease was statistically significant in 7 cancer types (*P* < 0.05; see Supporting Data Values file).

### Chromosome Y gain is associated with increased expression of Y-linked genes.

Using YchrRNA, YchrS scores, and matched tumor–normal data, we tested whether tumors with chromosome Y gain show elevated expression of Y-linked genes. Analysis of differential expression (log_2_[fold change]; log_2_FC) revealed 8 Y-linked genes to be significantly (false discovery rate–adjusted *P* values; *P*_FDR_) upregulated in Gain tumors versus normal tissue (*P*_FDR_ < 0.05, log_2_FC > 0): *TMSB4Y* (log_2_FC = 51.14, *P*_FDR_ = 0.0077), *DAZ2* (log_2_FC = 33.89, *P*_FDR_ = 0.0077), *TBL1Y* (log_2_FC = 13.16, *P*_FDR_ = 0.0158), *EIF1AY* (log_2_FC = 207.66, *P*_FDR_ = 0.0486), *NLGN4Y* (log_2_FC = 63.90, *P*_FDR_ = 0.0486), *DAZ1* (log_2_FC = 285.19, *P*_FDR_ = 0.0486), *SRY* (log_2_FC = 6.27, *P*_FDR_ = 0.0486), and *AMELY* (log_2_FC = 1.06, *P*_FDR_ = 0.0486). No Y-linked genes showed significant downregulation (Supplemental Figure 1 and Supporting Data Values file; supplemental material available online with this article; https://doi.org/10.1172/jci.insight.201996DS1).

### mLOY is associated with reduced PAR1 and PAR2 gene expression.

Pseudoautosomal regions (PAR1 and PAR2) are short regions of homology between the mammalian X and Y chromosomes and behave like an autosome during meiosis. Because *PAR1* and *PAR2* genes reside on both the X and Y chromosomes and normally require 2 active copies in males, we examined whether mLOY disrupts their dosage-sensitive expression. *PAR* genes escape X-inactivation and are therefore expected to show reduced transcript levels when the Y-linked allele is lost. To determine whether mLOY leads to measurable transcriptional consequences beyond canonical Y-linked genes, we quantified *PAR1*, *PAR2*, and combined *PAR* expression across tumors stratified by Y-chromosome status. Analysis revealed that mLOY is associated with significantly reduced *PAR1* (*P* < 0.001) and *PAR*-combined expression (*P* < 0.001) versus WT, and a weaker but detectable reduction in *PAR2* expression (*P* = 0.065) (Supplemental Figure 2, A, C, and E). Across the 6 Y-chromosome status groups — WT, mLOY, partial loss of Y (pLOY), remaining loss of Y (rLOY), chromosome Y gain (Gain), or mixed gain/loss (Gain_Loss) — significant differences in *PAR1* (*P* < 0.001), *PAR2* (*P* < 0.046, with WT showing the highest median and widest range), and combined *PAR* expression (*P* < 0.001) were observed (Supplemental Figure 2, B, D, and F).

### Age-associated changes in YchrFISH scores.

Analysis of age-associated changes in YchrFISH scores reveals a tissue-context-dependent relationship between aging and mLOY (Supplemental Figure 3). A significant negative correlation with age was observed in histologically normal tissues from men with cancer, both in organs distant from the tumor [NM(inCA), *r* = –0.447, *P* < 0.001] and in epithelium adjacent to cancer [NM(adCA), *r* = –0.306, *P* < 0.05], whereas no such association was detected in tissues from cancer-free men or in carcinoma samples.

### YchrFISH score decreases progressively from normal to cancer.

**Figure 3:**
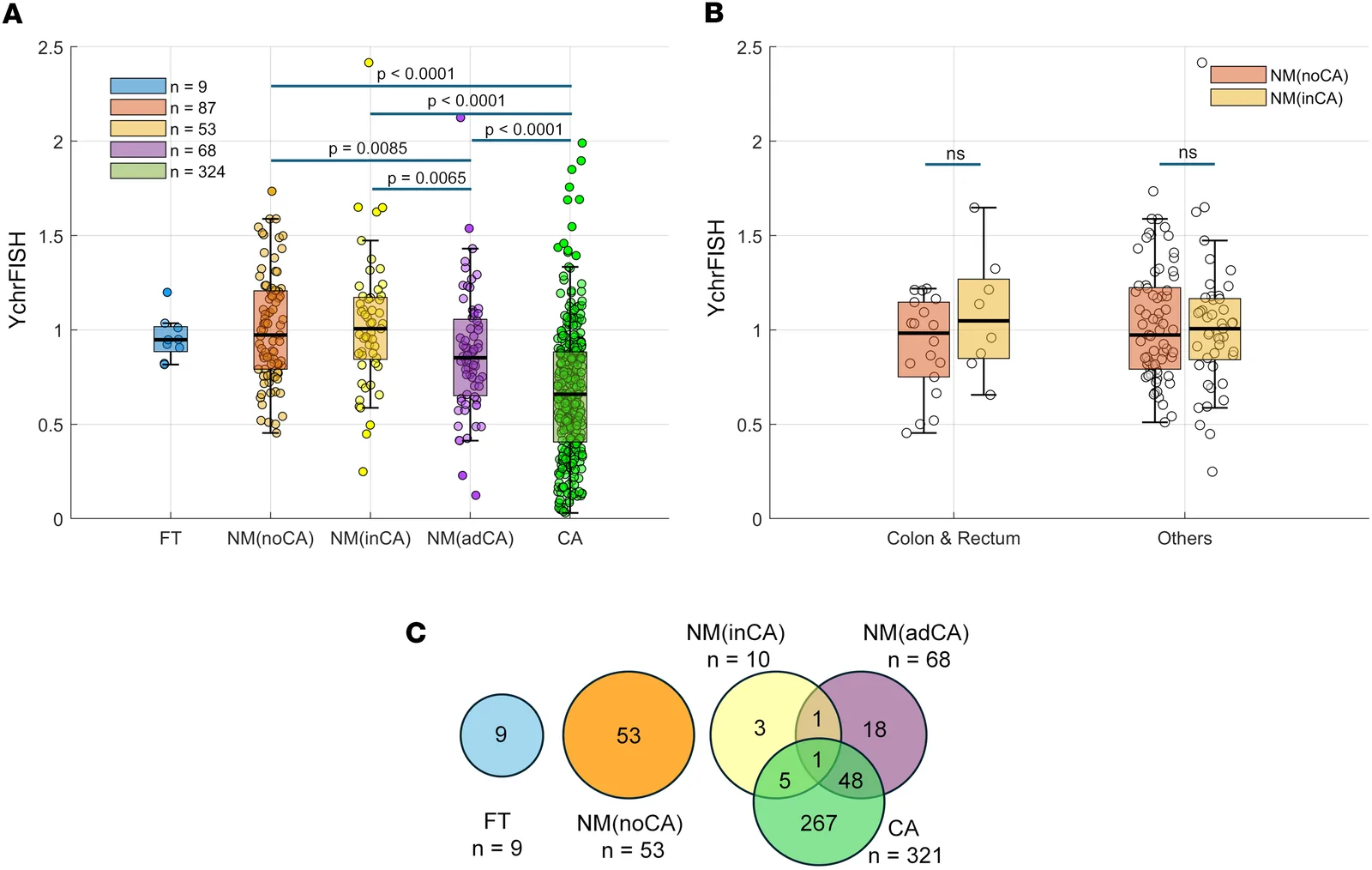
LOY increases progressively from normal to adjacent to malignant epithelium. (**A**) Distribution of organ-level FISH-derived LOY (YchrFISH) scores obtained from analysis of 1,000 TMA cores from 405 individuals (Table 1). One circle represents 1 organ-level YchrFISH score. Tissues are stratified into 5 types: fetal tissue (FT), histologically normal tissue from cancer-free individuals [NM(noCA)], histologically normal tissue from cancer patients sampled from a non-malignant organ [NM(inCA)], histologically normal epithelium adjacent to carcinoma [NM(adCA)], and carcinoma (CA). Statistical analysis was carried out using the Wilcoxon rank-sum test. (**B**) YchrFISH scores in NM(noCA) and NM(inCA) derived from colon and rectum (*n* = 18 and *n* = 8 individuals, respectively) juxtaposed with YchrFISH scores from 10 other organs (*n* = 69 and *n* = 45 individuals, respectively). The organ-level YchrFISH scores in NM(noCA) tissue and NM(inCA) tissue originate from 53 and 10 individuals, respectively. A summary of the number of analyzed samples, stratified by organ, tissue type and match status can be found in Supplemental Table 1. Statistical analysis was carried out using the Wilcoxon rank-sum test. (**C**) Venn diagram showing the distribution of tissue types across individuals (*n* = 405). FT, NM(noCA), NM(inCA), NM(adCA), and CA samples spanned major organs and included tissues from multiple organs obtained from the same individual. Among 321 men with cancer, 49 contributed paired same-organ CA and NM(adCA) tissue samples. Three of the 321 cancer-bearing men had cancer in 2 major organs resulting in 324 organ-level CA YchrFISH scores presented in Figure 3A. Additional paired NM(adCA) and CA samples (i.e., without paired tissue from the same organ) originated from 18 and 267 men, respectively. Fetal tissues from multiple organs (FT; *n* = 9 donors) served as reference groups in the analysis.

Using the YchrFISH score, we profiled 1,000 male tissue cores from 5 groups as mentioned above: FT (*n* = 9 male donors), NM(noCA) (*n* = 53 individuals), NM(inCA) (*n* = 10 individuals), NM(adCA) (*n* = 68 individuals), and CA (*n* = 321 individuals). YchrFISH scores were tightly centered near unity in FT and loosely centered at 1 in NM(noCA), consistent with a diploid baseline. The median levels of YchrFISH scores in NM(inCA) and NM(noCA) were similar, while NM(adCA) showed a significant decrease from NM(inCA), indicating increased mLOY in normal-appearing tissues adjacent to the tumor (Figure 3A). CA displayed the lowest YchrFISH scores with the broadest dispersion, consistent with strong mLOY enrichment in malignant epithelium. The median YchrFISH score differed significantly between NM(noCA) and NM(adCA) (*P* < 0.01), as well as between NM(inCA) and NM(adCA) (*P* < 0.01). In contrast, none of the comparisons between NM(noCA) and NM(inCA) overall or within individual organ types, differed significantly (Wilcoxon’s rank-sum test, *P* > 0.718) (Figure 3, A and B, and Supplemental Table 1; distribution of tissue types across individuals is shown in Figure 3C). These findings support a progressive increase in mLOY from normal through adjacent epithelium to carcinoma. Importantly, mLOY in cancer patients was limited to the organs with malignant transformation.

### mLOY is present in normal tissues adjacent to cancer.

**Figure 4:**
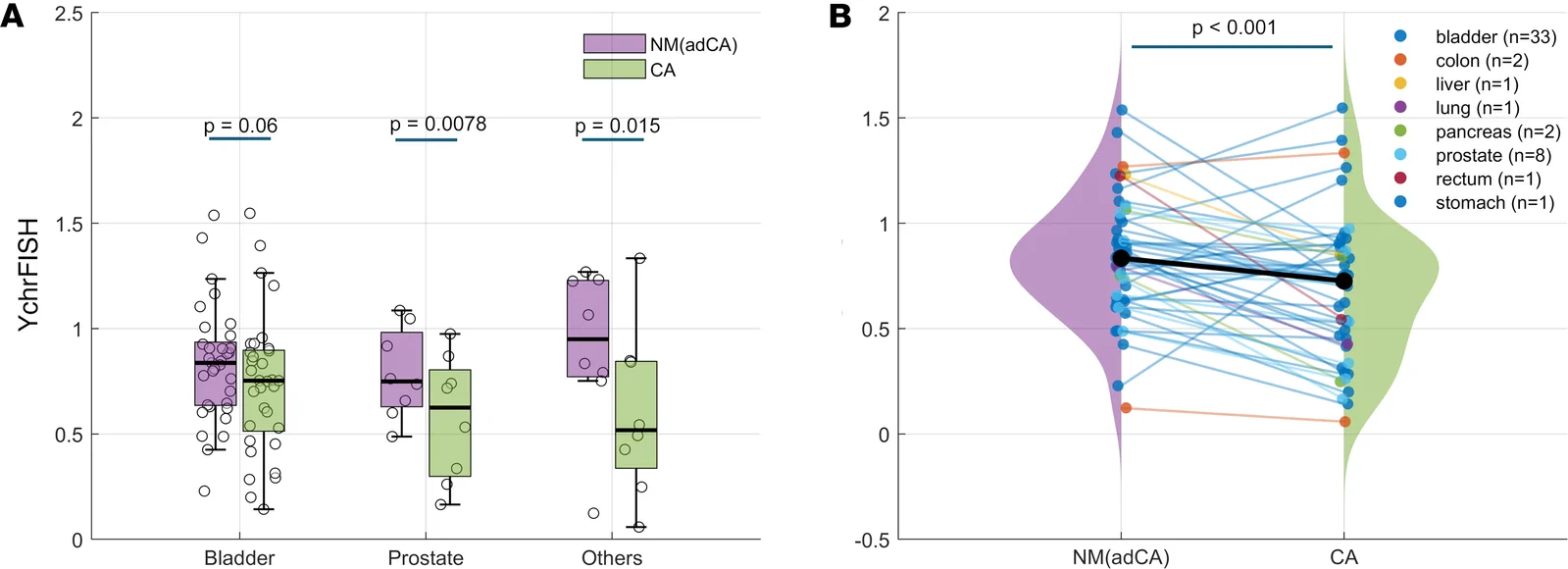
Comparison of YchrFISH scores between matching carcinoma (CA) and adjacent normal [NM(adCA)] across different organs. (**A**) Paired analysis (same organ) YchrFISH scores demonstrates lower YchrFISH scores in carcinomas than in their matched adjacent normal tissues, including bladder (*n* = 33 individuals), prostate (*n* = 8 individuals), and other organs (colon, liver, lung, pancreas, rectum, and stomach) combined (*n* = 8 individuals). Statistical significance was assessed using the Wilcoxon signed-rank test. Cancer types represented by ≤2 paired samples (colon, liver, lung, pancreas, rectum, and stomach) were combined into a single “other organs” group because individual statistical analyses were not informative. (**B**) Combined analysis of all paired samples (*n* = 49 individuals). Half-violin plots show the distribution of YchrFISH scores in NM(adCA) and CA samples. The overall difference between paired samples was significant (paired Wilcoxon signed-rank test, *P* = 0.00051). A summary of the number of analyzed samples, stratified by organ, tissue type, and match status can be found in Supplemental Table 1.

We further examined whether the degree of mLOY was elevated in histologically normal adjacent epithelium in different cancer types (Figure 4). In our TMAs, sufficient samples for tumor-type-specific analysis of tumor and adjacent normal tissue were only available for bladder and prostate carcinoma. Cancers with less than 5 samples (colon and rectum, lung, liver and bile duct, pancreas, esophagus and stomach) were pooled into an “Others” group. In this subset of samples, YchrFISH scores were consistently lower in CA compared with NM(adCA) (Figure 4A), indicating greater mLOY in tumor epithelium. The most pronounced separation was observed in prostate, with median YchrFISH scores of 0.749 in NM(adCA) and 0.624 in CA. The aggregated “Others” group showed a larger discrepancy, with median scores of 0.949 in NM(adCA) and 0.517 in CA, reflecting approximately equal detection of X and Y chromosomes in adjacent normal epithelium versus an approximately 50% reduction in Y chromosome detection relative to X in carcinomas. The smallest downward shifts were seen in bladder, with median NM(adCA) versus CA scores of 0.837 versus 0.752. Across all 8 organs, mLOY rates differed significantly between NM(adCA) and CA tissues in both paired samples (Figure 4B, *P* < 0.001) and the combined paired and unpaired dataset (Supplemental Figure 4; “Others”, *P* < 0.01). These findings suggest that the extent of mLOY in the “pre-neoplastic field” is not directly proportional to the degree of mLOY in the corresponding malignancies, suggesting that progressive accumulation of mLOY in normal cells is not uniform across organ types.

### Evidence for progressive Y-chromosome loss in transformation and progression.

**Figure 5:**
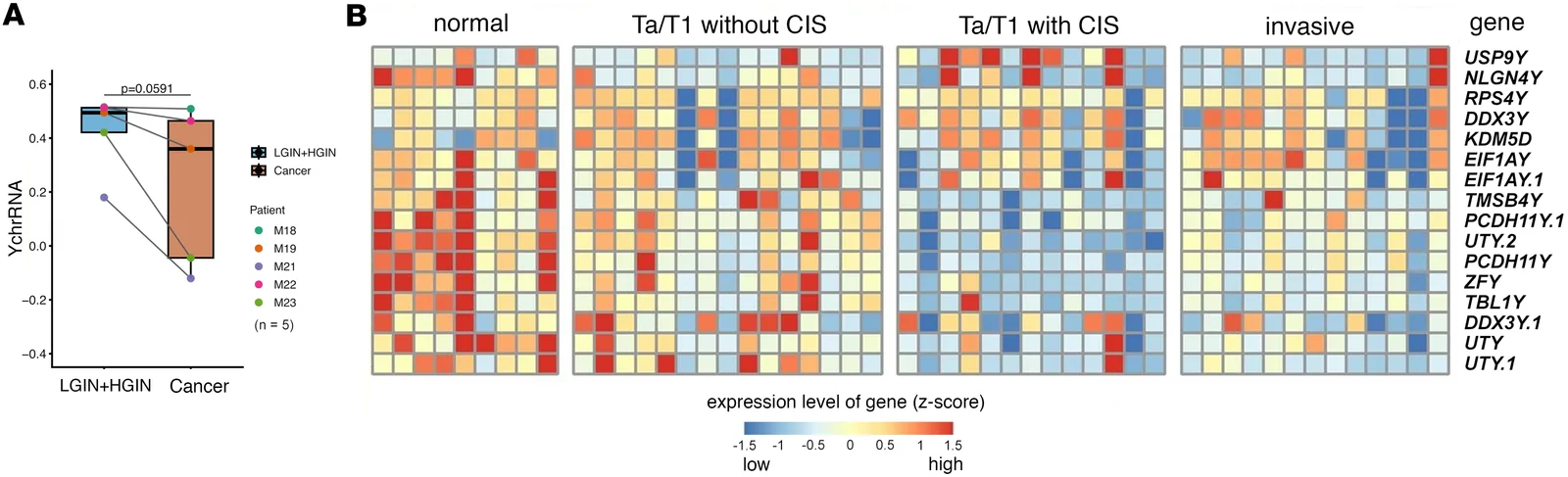
Reduced Y-chromosome RNA signature during urothelial transformation. (**A**) YchrRNA scores derived from spatially mapped low-grade (LGIN) and high-grade (HGIN) intraepithelial lesions matched with invasive bladder carcinoma (Cancer) in 5 individuals (M18, M19, M21, M22, and M23); data were retrieved from Bondaruk et al. and Majewski et al. (20, 21). Because matched, consistently annotated normal urothelium was not available in sufficient numbers from these same LGIN/HGIN cystectomy-map cases, the paired analysis was restricted to LGIN/HGIN and invasive carcinoma. Each dot represents the individual-level average YchrRNA score from the indicated color-coded pathological category: LGIN+HGIN (*n* = 58 and 43 samples) and Cancer (*n* = 15 samples). The analysis was performed using the paired Wilcoxon signed-rank test. (**B**) Heatmap of representative Y-linked genes in an independent cohort (23) including normal urothelium (*n* = 9), Ta/T1 tumors without carcinoma in situ (CIS) (*n* = 15), Ta/T1 tumors with CIS (*n* = 13); and invasive bladder cancers (*n* = 13), and each row to a Y-linked gene. Expression levels are shown as row-scaled *z* scores (red = higher expression, white = median, blue = lower expression).

To complement the FISH-based findings above, we leveraged whole-organ RNA-seq maps (20, 21) that profiled spatially resolved urothelial samples within individual cystectomy specimens. We applied a published Y-chromosome expression signature (22), together with curated Y-linked gene sets on these samples to systematically assess transcriptional surrogates of mLOY. YchrRNA scores in regions classified as histologically low-grade intraepithelial neoplasia (LGIN) and high-grade intraepithelial neoplasia (HGIN) were compared with urothelial carcinoma scores. This analysis revealed a decline in YchrRNA scores within the same organ, with male epithelium exhibiting higher LGIN+HGIN YchrRNA score values than in invasive carcinoma (*P* = 0.059) (Figure 5A).

These findings were consistent with expression data from Dyrskjøt et al. (23), which profiled bladder tumors and urothelium across pathological stage and the presence of CIS. Our reanalysis of these data revealed a coordinated downward shift in the expression of multiple Y-linked genes, including *USP9Y*, *DDX3Y*, *KDM5D*, *UTY*, *ZFY*, and *TBL1Y*. Heatmap visualization demonstrated that these transcripts attenuated in parallel with disease progression (Figure 5B). Together, these results extend our paired-core FISH-based analyses by showing that at the transcriptomic level mLOY progresses from noninvasive carcinoma to invasive disease. These findings support the concept that mLOY occurs in early lesions and is an escalating event in bladder tumor progression.

## Discussion

mLOY has been frequently observed in the blood of healthy aging men (1). mLOY has also been reported in skin (24), brain (25), bone marrow (26), cheek swabs (buccal cells) (1, 27, 28), and urine (29, 30) specimens. A recent scDNA-seq study of 102 nuclei from lung and colon tissues of a 74-year-old male presented enormous mLOY heterogeneity across organs and cells (31). The clinical relevance of these to patient outcomes and to blood mLOY remain to be determined. By showing an association between mLOY in histologically normal tissue adjacent to cancer but not in organs uninvolved with tumors, our pan-organ analysis provides the rationale for the concept that mLOY in non-tumorigenic tissues is an early event in transformation. Our work also supports the contention that mLOY is not a “passenger biomarker” of aging, but a direct pathogenic driver of age-related diseases in males (22, 32).

Three principal insights emerge that reshape our understanding of mLOY in oncogenesis. First, the current study reveals a stepwise accumulation of mLOY that parallels transformation and tumor progression. This was corroborated by independent transcriptomic analyses of spatially resolved bladder cystectomy maps supporting the presence of a continuous mLOY field effect during tumor evolution (20). Complementary differential expression analyses further showed that tumors classified as having chromosome Y gain exhibit significant upregulation of multiple Y-linked genes relative to matched normal tissues, confirming that increased Y-chromosome dosage translates directly into elevated Y-gene expression. Analysis of paired tumor-normal samples from TCGA revealed reduced expression of pseudo autosomal region genes (*PAR1* and *PAR2*) in tumors, consistent with consequences of Y-chromosome loss. Concordance between genomic (FISH) and transcriptional (RNA-seq) readouts supports the model of mLOY as a robust, early, and common somatic alteration in solid-tissue oncogenesis, defining a “pre-neoplastic LOY field effect” that precedes overt morphological malignancy (33, 34).

Second, our findings strongly suggest that this mLOY field effect has direct pathological consequences. mLOY in men with chronic kidney disease is associated with significantly increased risk of mortality and heart failure, identifying a high-risk subgroup for adverse cardiovascular outcomes (35). The present study expands this concept in oncology, revealing more mLOY in normal tissues adjacent to cancer compared with cancer-free individuals and normal tissue from individuals with cancer in another tissue. This suggests that a common pathogenic mechanism may be at play; Y chromosome–deficient immune or stromal cells, regardless of their location, may create a profibrotic (36) and proinflammatory microenvironment that is permissive for either tissue remodeling, as in the heart, or for malignant transformation.

Third, the striking tissue specificity of mLOY in cancer implies that local factors determine the ultimate pathological outcome of mLOY. While hematopoietic loss of Y chromosome leads to cardiac fibrosis and heart failure mortality (37), our current data show that within organs such as the bladder and prostate the primary consequence appears to be malignant transformation. This suggests that intrinsic, lineage-specific factors perhaps related to local immune surveillance or tissue-specific transcriptional networks modulate the response to mLOY, dictating whether the outcome is fibrotic disease or cancer. The enrichment of mLOY in tumors compared with adjacent normal tissue further argues that these clones are under active positive selection during carcinogenesis and progression.

Fourth, finding a negative correlation with age in histologically normal tissues from men with cancer either adjacent to the tumor or in organs distant from the tumor but not in men without cancer is conceptually aligned with finding that PBMC mLOY is associated with cancer development. We can speculate that age-related mLOY within histologically normal compartments is not a universal feature of male somatic tissues but preferentially manifests and drives cancer risk. The presence of age-associated mLOY in tissues distant from the tumor supports a systemic predisposition to Y-chromosome instability in cancer-bearing individuals, while its enrichment in tumor-adjacent normal tissue is consistent with field effects. In contrast, the lack of a detectable age correlation in carcinomas suggests that mLOY dynamics in tumors are shaped by clonal selection and additional genomic alterations, obscuring linear age-dependent trends. Together, these results support a model in which mLOY in PBMCs reflects genomic vulnerability linked to cancer risk by indirectly reporting the presence of mLOY in specific somatic tissues, the latter ultimately predisposing the cells to transformation. This model does not exclude the likely important contribution of specific PBMC populations with LOY to promote established progression once a tumor is established (32).

While our study is limited by its reliance on archival tissue from TMA slides with limited sample quantity, which may not always be representative of the entire heterogeneous tumor, the breadth of our analysis provides a crucial bridge between organ-specific pathology and systemic mosaicism. While no precursor lesions or malignant components were present in the normal tissue cores, including NM(adCA) and NM(inCA), we acknowledge that malignant cores contained normal stroma and inflammatory cells. As a result, the mLOY rate may be underestimated in malignant TMA cores due to the presence of non-malignant components. We also acknowledge that within normal samples, stromal, immune, and epithelial cells might have been present, potentially affecting the YchrFISH scores calculated for these samples. This critique is somewhat mitigated by the finding that normal cells infiltrating the tumor microenvironment also have mLOY (32). Our observation of mLOY occurring in early bladder lesions as an escalating event in bladder tumor progression warrants further investigation, given the small number of paired normal tissue samples. Future single-cell and spatial technologies will determine the relationships between LOY cells and their neighbors and how that relates to patient outcome.

In conclusion, by placing our pan-cancer findings in the context of prior mechanistic work on mLOY (20), we provide compelling evidence that positions mLOY in benign cells as a fundamental driver of male-specific carcinogenesis. This unified view provides a fresh perspective and working model of why aging men are more susceptible to cancer, establishing mLOY as a key therapeutic, diagnostic, and prevention target. These findings pave the way for targeted interventions, such as early detection followed by approaches to prevent Y-chromosome loss or eliminate normal or premalignant cells with mLOY.

## Methods

### Sex as a biological variable.

Because mLOY is a male-specific phenomenon, only tissues from individuals assigned male at birth were included in analyses of mLOY by immunofluorescence. Female samples were used only as negative controls to confirm assay specificity. Within the male cohort, analyses were stratified by organ. Sex was therefore an intrinsic biological variable in study design, and its consideration was essential both for assay validation and for result interpretation.

### TMA slide preparation, staining, and imaging.

This study analyzed 30 TMAs containing malignant (CA) and normal (NM) tissue samples collected from individuals with and without cancer. The TMAs were constructed using malignant and normal tissue sourced from the CSMC Biobank (autopsy and bladder; 13 TMAs), a commercial vendor (multiorgan and fetal tissue; 7 TMAs from https://tissuearray.com/), and collaborating institutions (multiorgan, prostate, and colon; 10 TMAs from USCF, UVA, and CHTN). Normal tissue cores were categorized into 4 groups: (a) NM(noCA), (b) NM(inCA) with NM and CA from different organs, (c) NM(adCA) from the same organs, and (d) normal FT, collectively accounting for 2,917 cores (Table 1). The TMAs were reviewed by an experienced genitourinary pathologist. No precursor lesions or malignant components were present in the normal tissue cores, including NM(adCA) and NM(inCA). Clinicopathological parameters, including sex (male or female), age, organ site, and tissue categories were cross-referenced with the YchrFISH scores for statistical analysis. Additionally, 2 rapid autopsy metastatic prostate cancer TMAs from the University of Washington (UW, 96 cores of metastases from 16 individuals across 15 metastatic sites collected by the Prostate Cancer Donor Program) and corresponding bulk RNA-seq data from paired OCT-frozen tumor samples (19) were used to validate the FISH assay (see *mLOY score quantification and validation*).

Freshly sectioned TMA slides (4 μm or 5 μm thick) were processed for FISH assay. Following deparaffinization, the TMA sections were stained using the AneuVysion Multicolor DNA Probe Kit (CE) (PROBE MIXTURE 1, Abbott Laboratories, 05J38-050), adhering to the manufacturer’s guidelines, and counterstained with DAPI. This kit employs FISH probes to detect α satellite DNA in the centromeres of chromosome Y (Yp11.1-q11.1, orange fluorescence) and chromosome X (Xp11.1-q11.1, green fluorescence) and is designed for karyotyping in amniotic fluid samples and FFPE specimens (38). TMA slide imaging was performed immediately after staining utilizing the ZEISS Axio Scan.Z1 scanner equipped with a Plan-Apochromat 20×/0.8 NA magnification objective and a Hamamatsu Orca Flash camera. The scanner captured 16-bit monochromatic images with the pixel size of 0.326 μm × 0.326 μm for each staining layer. The complete TMA slide image, including DAPI and FISH (orange and green) layers, was saved as a single.czi file.

### TMA slide image de-arraying.

The whole-slide TMA image was then loaded up and processed using the TMA de-array software tool (39) to automatically detect individual core images and assign unique coordinates to each core image. After manual inspection, corrections were made to add missing cores, remove cores with folded tissue, and improperly detected cores. Final core images were then extracted and saved as 3-layer (DAPI, green, orange) TIFF files. Coordinates of these images were cross-referenced with TMA documentation to exclude control and female tissue cores during downstream analysis.

### Core image quality control.

To identify FISH spots, the quality of staining in the green and orange core images was evaluated using the Rose contrast-to-noise (CNR) criterion. The CNR was calculated as CNR = (M_s_ – M_b_)/σ_b_, where M_s_ is the mean intensity of FISH spot pixels, and M_b_ and σ_b_ represent the mean and standard deviation of pixels within the nucleus, excluding the spot. According to this criterion, spots with a CNR below 3 to 5 are challenging to observe (15, 16). A similar calculation was performed for DAPI images. Here, nuclear pixel intensities used for M_s_ and pixel intensities outside the nucleus used for M_b_ and σ_b_. To evaluate the quality of the entire core image, CNRs were calculated separately for the orange, green, and DAPI layers using pixels from 10 randomly selected nuclei, each containing at least 1 FISH spot per color. The CNRs were then averaged within each layer. Core images were adequate for subsequent analyses only if the average CNR for each layer exceeded 5. Core images not meeting this criterion were excluded.

### Cell nuclei segmentation and FISH spot detection.

To automate mLOY quantification, we used previously developed image processing techniques to provide a nuclear mask, and a spot mask for tissue images stained using the FISH assay. For core images adequate for this analysis, the nuclear mask was obtained through StarDist, a publicly available neural network model that identified and delineated nuclei in the DAPI image (40). To obtain FISH spot masks, the Fast Radial Symmetry–based (FRST-based) FISH spot detector (17, 18) was applied separately to the orange and the green image. Prior to detecting spots, the orange and green images were preprocessed by the Gaussian filter to flatten the background, and subsequently by the anisotropic diffusion filter (41) to attenuate debris and nonspecific FISH staining. By providing a range of spot radii (in pixels), the FRST-based detector detected spots with matching radii. The range of matching radii was established based on the minimum (~0.35 μm, 1 pixel) and the maximum (~0.78 μm, 2 pixels) spot radius found in the orange and green images. Additionally, by setting up the radial strictness parameter α to 2, FRST detected circular spots and omitted oval and elongated spots that would require a higher α value set for their detection. As a result, FRST outputs an intensity image with peaks indicating the positions of detected spots. Thresholding this image using a cutoff value calculated as described in Gertych et al. and Bahry et al. (17, 18) yielded a binary spot mask. For visualization, the detected spots were marked by crosshairs (Figure 1, C right column, and D inset).

### mLOY score quantification and validation.

The FISH-assay based YchrFISH score was calculated for each TMA core as the ratio of orange to green FISH spots detected under the nuclear mask. To evaluate adequacy of the FISH assay for mLOY quantification in our TMAs, the FISH-based mLOY scores were compared to the bulk RNA-seq–based mLOY scores from OCT-frozen tumor samples in metastatic prostate cancer specimens from UW (19). A total of 77 TMA core images from 25 tumors passed quality control and were used for regression analysis. The YchrFISH scores of TMA cores punched from the same specimen/tumor were averaged. The null hypothesis was that there is no significant association between the FISH- and RNA-seq–based mLOY scores.

### Gene-level analysis.

To compare YchrFISH scores with RNA-seq–based mLOY scores (YchrS), we analyzed primary tumor samples and paired tumor and matched normal samples from male-proxy primary tumors in the TCGA Pan-Cancer RNA-seq matrix (42). To perform the analysis, we defined the YchrS score as the mean log_2_ expression of the 9 Y-chromosome genes (*DDX3Y*, *UTY*, *KDM5D*, *USP9Y*, *ZFY*, *RPS4Y1*, *TMSB4Y*, *EIF1AY*, and *NLGN4Y*) from Chen et al. (32) and analyzed cases spanning 28 cancer types. Male samples were determined based on a YchrS score cutoff of 0.5 or greater. For the paired analysis, we identified those male individuals who had both a tumor and a matched normal sample, spanning 19 cancer types and calculated ΔYchrS as tumor YchrS minus paired solid-tissue normal YchrS.

Additionally, analysis of RNA expression of *PAR1* and *PAR2* (pseudoautosomal region) genes that are present on both the X and Y chromosomes was performed to assess whether the expression of this gene set is altered in mLOY tissues. To directly test whether loss of the mLOY alters expression of *PAR* genes — which reside on both X and Y chromosomes and are expected to be dosage sensitive in males — we quantified RNA expression of *PAR1* and *PAR2* gene sets in the TCGA cohort using the same bulk RNA-seq matrix employed for the YchrRNA/YchrS analyses. For this analysis, we defined *PAR1* and *PAR2* scores as the mean log_2_ expression of each gene, and the *PAR* combined score was defined as the mean expression of *PAR1* and *PAR2* genes. Samples were stratified by tumor Y-chromosome status into 6 categories: WT, mLOY, pLOY, rLOY, Gain, or Gain_Loss, as determined by the whole-genome sequencing (WGS) and FACETS copy-number analysis adopted from Qi et al. (9) (Supplemental Methods).

### Spatial RNA-seq dataset acquisition.

Spatially resolved bladder RNA-seq data were obtained from the cystectomy map resource of Bondaruk et al. and Majewski et al. (20, 21), which provides grid-defined transcriptomic profiles across histologically annotated urothelial regions. Each sample is annotated as LGIN or HGIN, or invasive carcinoma (Cancer), and enabling within-organ comparisons between LGIN+HGIN versus Cancer that minimize interpatient variability.

### Bladder cancer dataset.

We reanalyzed expression profiles from the bladder cancer microarray dataset from 5 individuals reported by Dyrskjot et al. (23). This dataset includes samples spanning histologically normal urothelium, CIS, superficial tumors, and muscle-invasive tumors. Probes corresponding to Y-linked genes (*USP9Y*, *DDX3Y*, *KDM5D*, *UTY*, *ZFY*, *TBL1Y*, and others) were extracted, normalized, and visualized as heatmaps as previously described (32). Expression heatmaps were generated using variance-stabilized counts, with rows representing genes and columns representing samples ordered by pathological stage (normal → Ta/T1 without CIS → Ta/T1 with CIS → Invasive Cancer). Relative expressions were scaled per gene, and color values represent standardized expression (red = higher, blue = lower, white = median).

### Statistics.

All statistical analyses of YchrFISH scores were performed at the individual level. When multiple tissue cores were available from the same individual and organ, values were averaged prior to analysis. Paired statistical tests were used when comparing different tissue types obtained from the same individual. Group comparisons were performed using the Wilcoxon rank-sum test or paired Wilcoxon signed-rank test. The Kruskal-Wallis test was used to evaluate differences between 3 or more independent groups. All tests were 2-sided, and statistical significance was defined at a threshold of *P* less than 0.05 unless otherwise specified. Correlation analyses were conducted using Pearson’s correlation and *t* test. Violin and half-violin plots were used to visualize YchrFISH score distributions. Statistical significance in differential gene expression analysis was determined using Wald’s test, and *P* values were corrected for multiple testing using the Benjamini-Hochberg (BH) FDR, with adjusted *P* values (*P*_FDR_) reported throughout. The BH *P*-value adjustment was carried out based on their rank rather than their absolute magnitude. Hence, genes with similar or identical raw *P* values received the same *P*_FDR_ value. The BH method produced stepwise adjusted values, which can cause groups of genes — especially those with similar expression patterns — to share identical corrected *P* values. Genes with *P*_FDR_ less than 0.05 were considered significantly differentially expressed. Log_2_FC values were used to quantify differential gene expression, with positive log_2_FC indicating upregulated genes and negative log_2_FC indicating downregulated genes. Linear regression modeling was used to assess significance of association between the Y-chromosome expression score (YchrRNA) and the FISH-based mLOY score (YchrFISH), and to visualize correlation between YchrFISH and age across different tissue types. Significance was assessed using a *t* test. In box-and-whisker plots, boxes indicate the interquartile range (IQR), horizontal lines within boxes denote the median, and whiskers extend to 1.0 × IQR.

### Study approval.

The data collection and analysis were approved for research by the Cedars-Sinai Institutional Review Board under project number Pro000043021.

### Data availability.

The data supporting the findings of this study are available within the article. Values for all data points in graphs and average FISH score values per case/tumor are reported in the Supporting Data Values file. Supporting analytic code is available upon request.

## Author contributions

DT conceptualized the idea and hypothesis and initiated the study. AG, HY, XC, EV, and VKR developed the methodology, acquired data, performed analyses, and generated graphs. HY provided pathological expertise and reviewed TMAs. LB, LDT, PSN, and PRC provided reagents and advice. AG, HY, XC, and DT wrote and revised the manuscript.

## Conflict of interest

The authors have declared that no conflict of interest exists.

## Funding support

This work is the result of NIH funding, in whole or in part, and is subject to the NIH Public Access Policy. Through acceptance of this federal funding, the NIH has been given a right to make the work publicly available in PubMed Central.

- National Cancer Institute grant R35CA294022 to DT.
- Pacific Northwest Prostate Cancer SPORE grant P50CA97186.
- NIH program project grant P01CA163227 to PSN.
- Institute for Prostate Cancer Research grant to LDT and PSN.

## Supplementary Material

Supplemental data

Supporting data values

## Figures and Tables

**Table 1:**
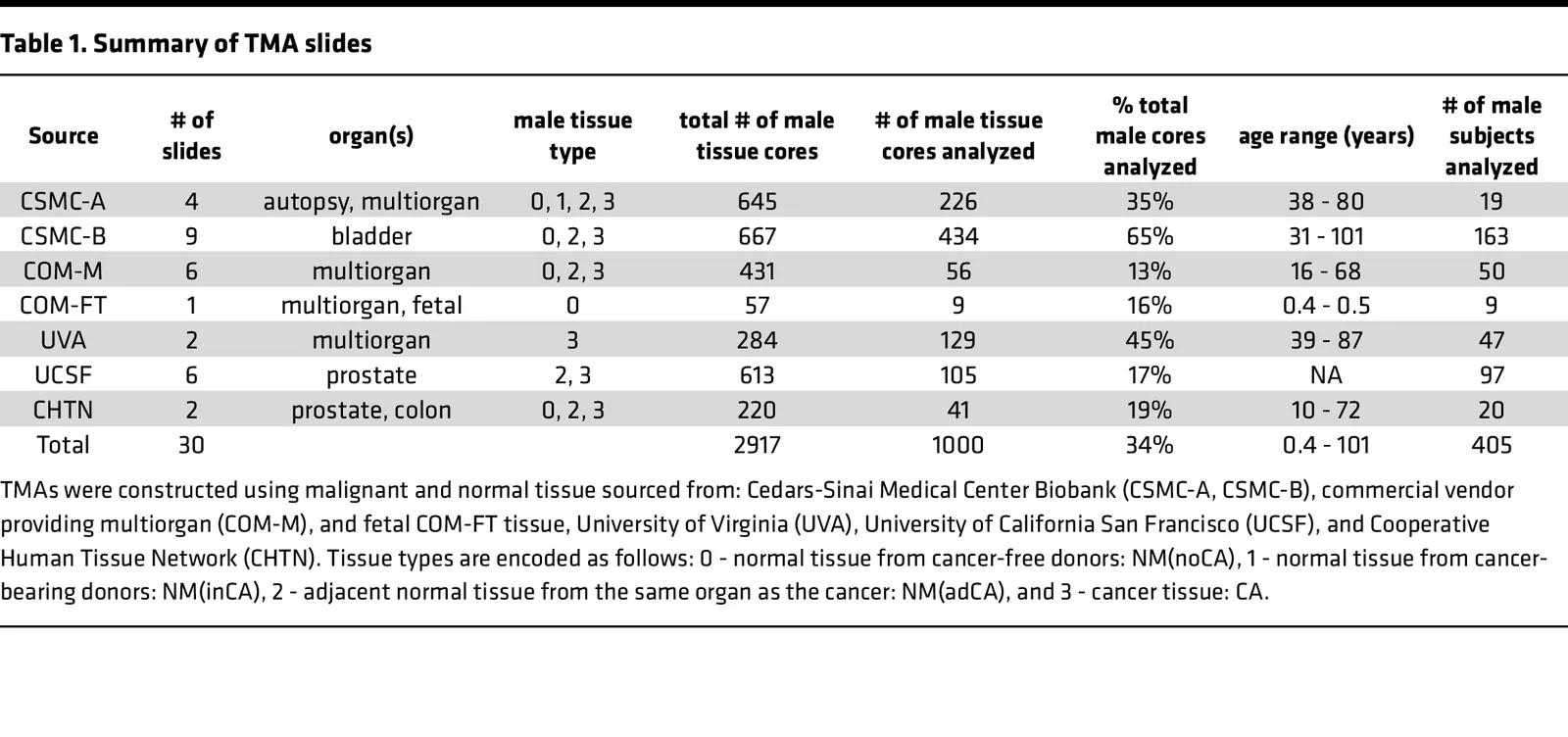
Summary of TMA slides

## Version 1 09/08/2026

Electronic publication
