# Supplementary material for: Human Y chromosome pan-organ mapping reveals progressive mosaic loss from normal to cancer
Source: JCI Insight. 2026 Sep 8;11(17):e201996. doi: 10.1172/jci.insight.201996 (PMC13564088; doi:10.1172/jci.insight.201996)
Supplement: Supplementary file 1 [file jciinsight-11-201996-s084.pdf]

Human Y chromosome pan-organ mapping reveals progressive mosaic loss from normal to cancer

Arkadiusz Gertych[1,2#], Huihui Ye[2#], Xingyu Chen[3,4], Eric Vail[2], V. Krishnan Ramanujan[2], Lauren Brady[5], Lawrence True[6], Peter S. Nelson [5,6], Peter R. Carroll[7],
Dan Theodorescu[2,3,8*]

Supplemental: Methods, Tables and Figures

Supplemental Methods

Y chromosome copy-number status was classified following the strategy described by Qi et al. in (9). Briefly, total copy number (tcn) of Y chromosome segments was estimated, and the expected wild-type Y copy number was defined according to tumor ploidy. For samples with odd rounded ploidy, the expected maximum Y wild-type copy number was defined as ceiling(ploidy/2) and the expected minimum as floor(ploidy/2); for samples with even rounded ploidy, the expected Y wild-type copy number was defined as round(ploidy/2). A Y segment was classified as gained if tcn > max.Y.WT, lost if tcn < min.Y.WT or tcn = 0, and wild type (WT) if min.Y.WT ≤ tcn ≤ max.Y.WT.

Based on these segment-level calls, samples were further assigned to six categories: WT, when all Y segments were WT; Gain, when at least one Y segment was gained and all remaining segments were WT; Gain+Loss, when at least one segment was gained and at least one segment was lost; pLOY (partial loss of Y), when at least one segment was lost and all remaining segments were WT; rLOY, when ≥ 99% of the Y chromosome was lost but tcn ≥ 1; and LOY, when ≥ 99% of the Y chromosome was lost and tcn = 0. This classification scheme was adapted from the method reported in (9).

Supplemental Tables

Table 1. Number of samples, stratified by tissue type, organ and match status. Unpaired NM(noCA) and NM(inCA), or NM(adCA) and CA samples were obtained from different subjects, and paired NM(adCA) and CA samples were obtained from the same subject. Cell colors correspond with boxplot and Venn diagram colors in Figure 3 A, B, and C and with boxplot colors in Figure 4 A, and B, Supplemental Figure 4, and line and data point colors in Supplemental Figure 3. Sample quantities in the last column (Total) match the quantities in Figure 3A legend**,** and the numbers of paired samples match the number of samples in Figure 4B**.**

Tissue type	Number of samples per organ								Number of samples		Total
	Bladder		Prostate		Colon & Rectum		Others				
	paired	unpaired	paired	unpaired	paired	unpaired	paired	unpaired	paired	unpaired	
NM(noCA)	NA	*	NA	*	NA	18	NA	69	NA	87	87
NM(inCA)	NA	*	NA	*	NA	8	NA	45	NA	53	53
NM(adCA)	33	1	8	14	*	*	8	4	49	19	68
CA	33	122	8	89	*	*	8	64	49	275	324

* Samples from organs with number of CA or NM(adCA) samples < 5, or number with NM(noCA) or NM(inCA) samples < 5 are included in Others category. NA - not applicable.

Supplemental Figures

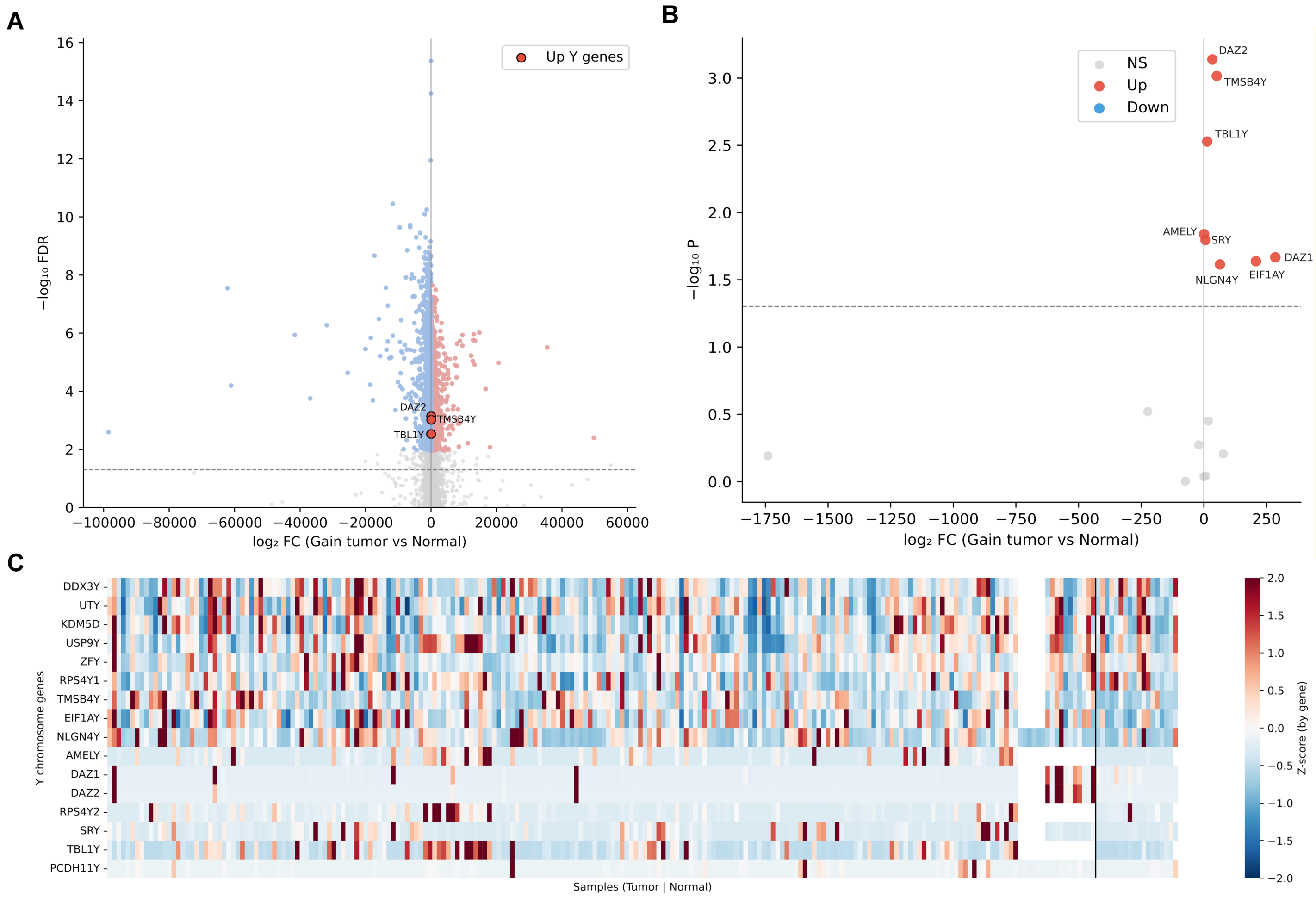

Supplemental Figure 1. Chromosome Y-linked gene expressions in a subset of samples from TCGA tumors. Tumors classified as having chromosome Y gain (Gain tumors; n ≈ 210) were compared with matched normal tissues (n = 18) using the TCGA Pan-Cancer bulk RNA-seq dataset. Analyses included the full complement of Y-chromosome genes, incorporating the nine signature genes described by Chen et al. in (32) together with additional Y-linked genes (e.g., *AMELY*, *DAZ1/2*, *SRY*, *TBL1Y*). **(A)** Genome-wide volcano plot highlighting Y-chromosome genes (red), demonstrating their enrichment among upregulated transcripts in Gain tumors. **(B)** Volcano plot restricted to 16 Y-chromosome genes in panel (C), with eight genes significantly upregulated (P_{FDR} < 0.05, Wilcoxon rank-sum test.). **(C)** Z-score heatmap of Y-chromosome genes across Gain tumor and normal samples, revealing consistent blocks of elevated expression in Gain tumors. Normal samples are divided from tumor by vertical black line.

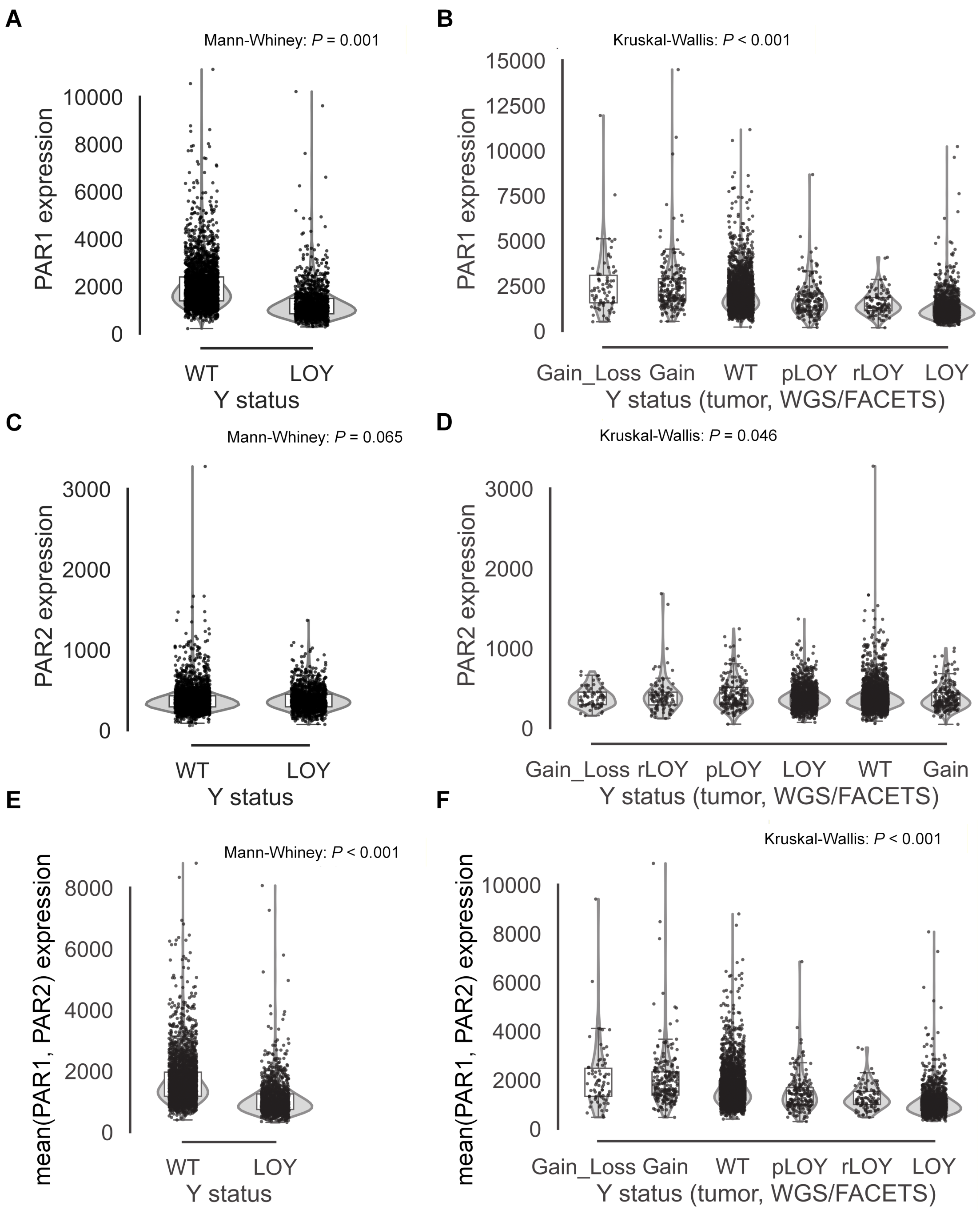

Supplemental Figure 2. *PAR1*, *PAR2*, and combined *PAR*-gene expression across tumor Y-chromosome states in the TCGA cohort. RNA expression of *PAR1* and *PAR2* gene sets was quantified using the TCGA bulk RNA-seq dataset employed for the YchrRNA/YchrS analyses (Figure 2, B–D). Samples were stratified by tumor Y-chromosome status as determined by WGS/FACETS (Figure 1). The right column shows comparison between two groups (WT and LOY), and the left column shows stratification into six groups (Gain_Loss, Gain, WT, pLOY, rLOY, LOY). **(A–B)** *PAR1* expression, **(C–D)** *PAR2* expression, and **(E–F)** combined *PAR* expression. *PAR1* and *PAR2* scores represent mean log2 gene expression values, and the combined *PAR* score reflects the sum of *PAR1* and *PAR2* scores. Statistical significance between WT and LOY was assessed using the Mann–Whitney U test (equivalent to Wilcoxon rank-sum test), and differences across the six Y-chromosome status groups were evaluated using the Kruskal–Wallis test. Plots are ordered by decreasing median expression level.

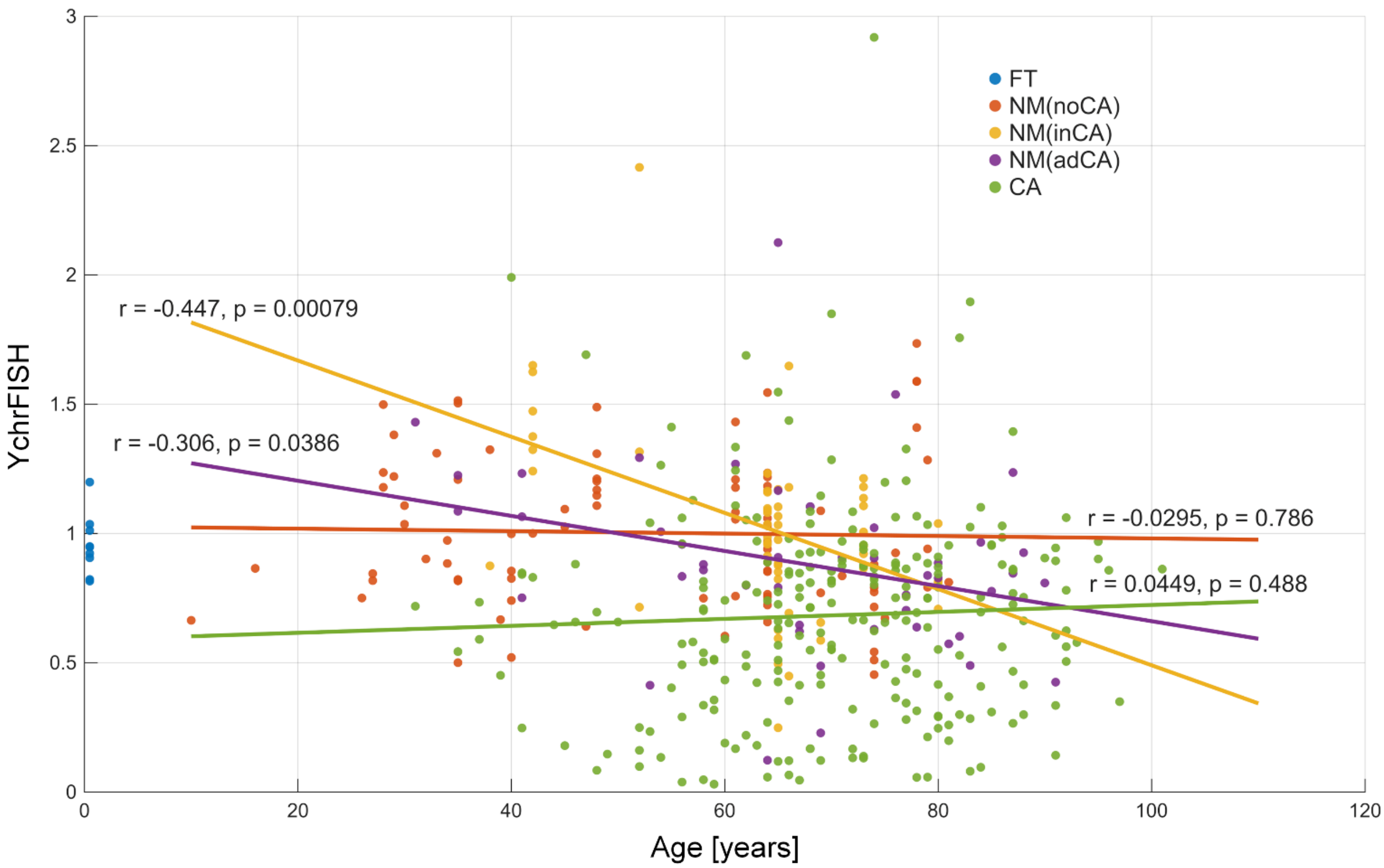

Supplemental Figure 3. Correlation of YchrFISH scores with age across tissue types. YchrFISH scores, reflecting loss of chromosome Y, were quantified in histologically normal and cancerous tissues obtained from multiple organs in 405 subjects. Tissue categories included histologically normal tissue from fetal tissue (FT) cancer-free men (NM(noCA)), histologically normal tissue from men with cancer in a different organ (NM(inCA)), histologically normal epithelium adjacent to cancer (NM(adCA)), and carcinoma (CA). Each data point represents the average YchrFISH score for one subject and one organ; when multiple samples were available from the same subject and organ, values were averaged. Samples from different organs within the same subject are shown as separate data points. Trend lines were fitted using linear regression models for each tissue type (excluding FT), and correlations with age were assessed using the Pearson correlation coefficient. A significant negative correlation with age was observed in NM(inCA) and NM(adCA) samples, whereas correlations were not significant in NM(noCA) or CA samples. Significance was assessed using t-test.

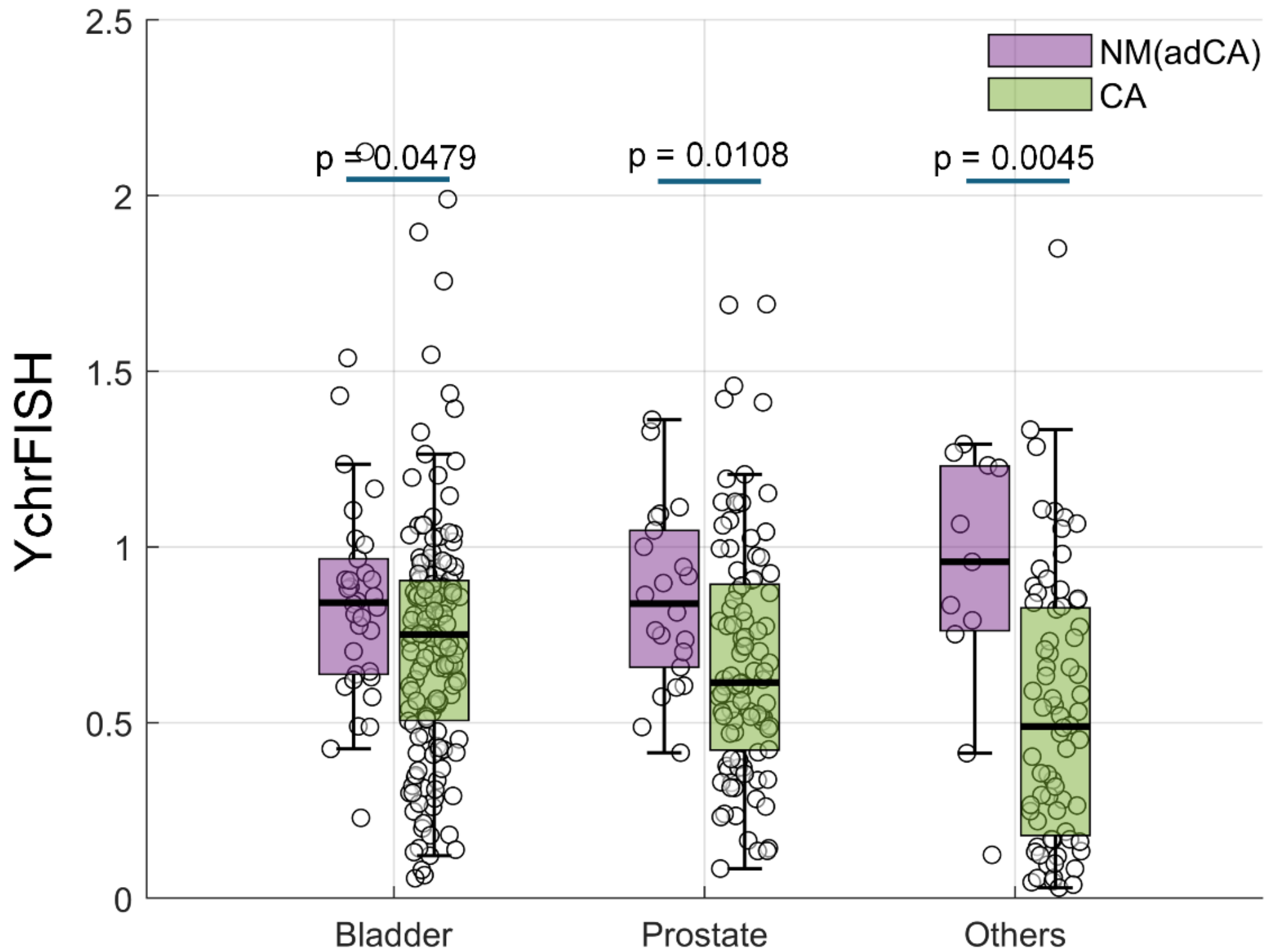

Supplemental Figure 4. Comparison of YchrFISH scores between carcinoma (CA) and adjacent normal (NM(adCA)) across different organs using paired (same organ and same subject) and unpaired (different subjects) samples combined. Analysis YchrFISH scores shows lower LOY scores in cancer compared to their matched normal counterparts in bladder (n = 34 (NM(adCA)), and n = 155 (CA) samples), prostate (n = 22 (NM(adCA)), and n = 97 (CA) samples), and other organs combined (n = 12 (NM(adCA)), and n = 72 (CA) samples). Analyses were carried out using Wilcoxon rank-sum test. A summary of the number of analyzed samples, stratified by organ, tissue type and match status can be found in Supplemental Table 1.
